# Loperamide-Induced Torsades de Pointes

**DOI:** 10.7759/cureus.20299

**Published:** 2021-12-09

**Authors:** Emmanuel Isang, Laylan Shali, Charles B Morris, Jeremy Mahlow

**Affiliations:** 1 Cardiology, University of Tennessee Medical Center, Knoxville, USA; 2 Internal Medicine, University of Tennessee Medical Center, Knoxville, USA

**Keywords:** medical toxicology, arrythmia, cardiac arrythmia, torsades de pointes, loperamide toxicity, loperamide

## Abstract

Loperamide, an over-the-counter antidiarrheal, works on the µ opioid receptor with minimal opioid activity if taken as directed. Recently, it has gained popularity as the “poor man's methadone” at supratherapeutic dosing. Opioid antagonism with naloxone is beneficial in reversing respiratory depression but has no effect on cardiotoxicity due to the human ether-a-go-go-related gene (hERG). We present the case of a 34-year-old female who presented for syncope after taking 48 tablets of 2 mg loperamide. On arrival, she was obtunded with variable heart block and a QTc of 560 ms. Subsequently, due to further QT prolongation from loperamide to 656 ms, she developed Torsades de Pointes requiring defibrillation at 120 J twice. Ultimately, she was discharged home with psychiatric and substance abuse outpatient follow-up. Patients and healthcare providers face new challenges with the increase in loperamide misuse due to easy access and delayed identification. It is important for clinicians to recognize and be familiar with loperamide overdose given the potential for multiorgan failure and increased mortality.

## Introduction

Loperamide is a µ opioid receptor agonist used to treat acute diarrhea of various etiologies. It stimulates the opiate receptors in the intestinal tract leading to decreased peristalsis [1]. Due to its poor bioavailability, rapid first-pass hepatic metabolism, and low central nervous system penetration at therapeutic doses (<16 mg daily), loperamide is presumed to have limited abuse potential.  This is largely due to P-glycoprotein pump that regulates the transportation of loperamide at the gastrointestinal tract and the blood-brain barrier [1,2]. At higher doses (100-400 mg), however, loperamide can cross the blood-brain barrier and enter the central nervous system to achieve euphoria or avoid withdrawal [3]. With the ongoing opioid pandemic, low cost, and easy accessibility, loperamide abuse has rapidly increased and has been known widely as the “poor man's methadone” [1-3]. We present a case of a 34-year-old female who presented with prolonged QTc after being found down secondary to taking 48 tablets of 2 mg loperamide. 

## Case presentation

**Figure 1:**
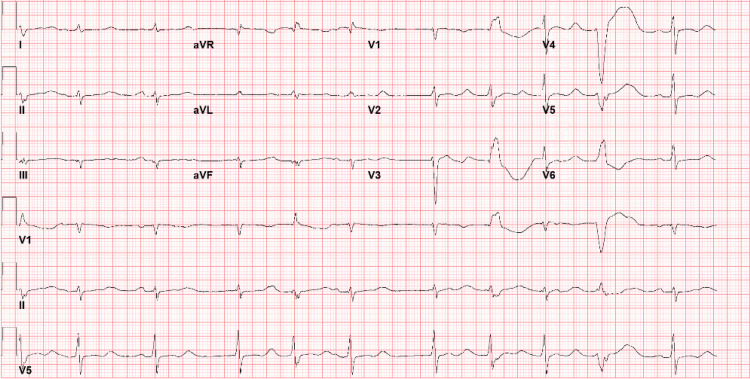
ECG on admission showing sinus rhythm with prolonged QT interval and PVC ECG: electrocardiogram, PVC: premature ventricular contraction

**Figure 2:**
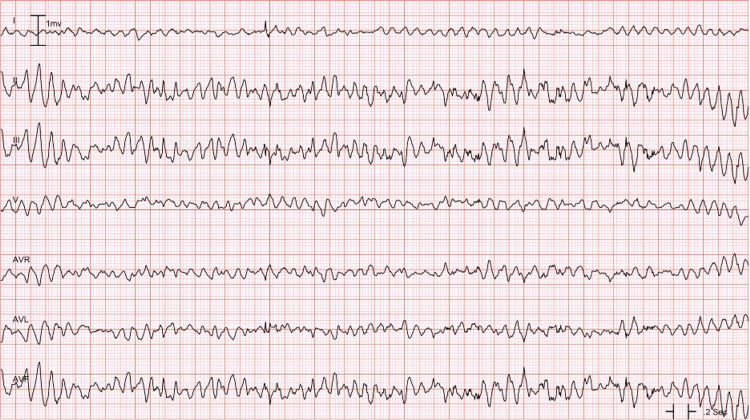
ECG showing Torsades de Pointes ECG: electrocardiogram

A 34-year-old female with a history of remote intravenous drug abuse with opiates, hepatitis C, and depression presented to the hospital after being found down and unresponsive. Duration of unresponsiveness was unknown as she was found by a family member with an empty bottle of over-the-counter (OTC) loperamide. Patient had previously taken excessive amounts of loperamide in hopes of obtaining euphoria and preventing withdrawal symptoms. Emergency medical services was called and she was transported to the emergency department (ED) for further evaluation. In the ED, electrocardiogram (ECG) revealed first-degree atrioventricular block with a heart rate of 86 beats per minute and a prolonged QTc of 560 ms (360-460 ms). Poison control was informed, who recommended initial treatment with naloxone. In addition, patient was given 6 mg of intravenous (IV) magnesium, 100 mEq of bicarbonate followed by a continuous bicarbonate infusion. Unfortunately, she became increasingly lethargic, hypoxic, and bradycardic with heart rate in the 30s. Repeat ECG revealed further prolongation of the QTc to 656 ms (Figure 1), and she was subsequently intubated for airway protection. She was immediately transferred to the intensive care unit and started on 3% hypertonic saline as well as lipid emulsion therapy. A few hours later, she went into Torsades de Pointes and was successfully defibrillated with 120 J (Figure 2). She was subsequently extubated the following day and closely monitored in the intensive care unit for an additional 24 h. Potassium, magnesium, and calcium levels were monitored and appropriately repleted.

Our patient continued to have frequent ventricular ectopy on telemetry. She became increasingly bradycardic followed by asystole and Torsades de Pointes for the second time. ECG revealed a QTc of 519 ms. Chest compressions were initiated, and she was successfully defibrillated once again using 120 J. She was given 2 g of IV magnesium and started on a continuous isoproterenol infusion at 5 mcg/min. She remained in the intensive care unit for several days until her QT prolongation resolved, with the last ECG showing a QTc of 455 ms. Isoproterenol infusion was successfully titrated down and stopped. She had no further ectopy on telemetry and was stable for discharge. Prior to discharge, patient was seen by psychiatry and deemed stable for discharge with close follow-up as outpatient. 

## Discussion

Loperamide is a µ opioid receptor agonist used mainly as an OTC antidiarrheal medication. At a therapeutic level, it has trivial central nervous system activity due to its low oral bioavailability and poor blood-brain barrier penetration. When first introduced in 1977, it was placed as a schedule V of the US Controlled Substances Act due to concerns related to its opioid effects. This was eventually removed due to lack of evidence showing physical dependence [4]. Following removal from schedule status and increasing opiate epidemic, rates of abuse have significantly increased. When used at higher-than-recommended doses or co-ingested with a P-glycoprotein inhibitor, it can result in euphoria and alleviation of withdrawal symptoms [5]. Unfortunately, loperamide ingestion can lead to cardiotoxicity, mainly due to inhibition of voltage-gated sodium/potassium channels, leading to QTc prolongation. Cardiotoxicity associated with the QTc prolongation includes wide complex tachycardias, polymorphic ventricular tachycardia, and sudden cardiac death [6]. The mainstay of treatment for loperamide toxicity is supportive care. In individuals with cardiotoxicity, treatment ranges from advanced cardiac life support, electrical pacing, or isoproterenol for suppression of ventricular ectopy. Long-term patients benefit from referral to and follow-up with addiction treatment programs [4-7]. 

Treatment of loperamide toxicity can vary based on patient presentation, with supportive therapy largely being the mainstay of therapy. Acute toxicity can be treated with activated charcoal within 2-4 h of ingestion [4]. Respiratory depression is a major concern with any excess opiate use. If there is concern for airway compromise, naloxone can be used preferably at the lowest effective dose to avoid any opiate withdrawal symptoms. Patients should still be closely monitored even if naloxone proves effective as loperamide can have a prolonged half-life [2]. 

 As seen in this case, QTc prolongation is a significant concern regarding loperamide toxicity. Initially, patients should be evaluated for any reversible factors for QTc prolongation, such as electrolyte abnormalities like hypokalemia and hypomagnesemia. Unlike traditional treatment of Torsades de Pointes, studies have shown that Torsades secondary to loperamide toxicity may not respond well to typical therapies with magnesium or sodium bicarbonate [2]. Thus, treatment may require cardiac pacing, electrical cardioversion, or IV isoproterenol to suppress ventricular ectopy and prevent arrhythmias from reoccurring [2]. If associated ventricular dysrhythmias occur, such as polymorphic ventricular tachycardia, treatment with magnesium sulfate and sodium bicarbonate has been successful [2]. Lipid emulsion therapy can serve a role for reversal of toxicity due to overdose. Though the mechanism of action is not completely understood, it is believed to be due to increased binding of loperamide to the lipid-rich emulsion, thereby drawing it away from central nervous and gastrointestinal system [8]. In addition, hypertonic saline is an effective treatment strategy by increasing the sodium concentration to overcome the sodium channel blockade caused by loperamide [9]. Ultimately, in the event of loperamide cardiotoxicity causing cardiopulmonary arrest, cardiopulmonary resuscitation and advanced cardiac life support should be considered as first-line treatment [2-4]. 

## Conclusions

Loperamide is a safe OTC antidiarrheal when used at therapeutic doses. Toxicity secondary to intentional and unintentional overdoses is increasingly frequent to both medical and poison control centers. Treatment is mainly supportive; however, it can be escalated depending on the clinical presentation. Loperamide is not normally included in drug screens and routine reporting is not required. This leads to delayed diagnosis, decreased awareness, and increased mortality. It is projected to increase in the absence of regulation, and with low cost and vast availability. Medical personnel should have increased awareness of loperamide misuse with patients who have opioid-like toxicity with prolonged QT interval. Patients who survive face increased length of stay and benefit from follow-up with an addiction treatment program. 
